# An insight, at the atomic level, into the polarization effect in controlling the morphology of metal nanoclusters†

**DOI:** 10.1039/d1sc00632k

**Published:** 2021-07-13

**Authors:** Xi Kang, Xiao Wei, Shuxin Wang, Manzhou Zhu

**Affiliations:** Department of Chemistry, Centre for Atomic Engineering of Advanced Materials, Anhui Province Key Laboratory of Chemistry for Inorganic/Organic Hybrid Functionalized Materials, Anhui University Hefei 230601 P. R. China ixing@ahu.edu.cn zmz@ahu.edu.cn; Key Laboratory of Structure and Functional Regulation of Hybrid Materials, Anhui University, Ministry of Education Hefei 230601 P. R. China

## Abstract

The polarization effect has been a powerful tool in controlling the morphology of metal nanoparticles. However, a precise investigation of the polarization effect has been a challenging pursuit for a long time, and little has been achieved for analysis at the atomic level. Here the atomic-level analysis of the polarization effect in controlling the morphologies of metal nanoclusters is reported. By simply regulating the counterions, the controllable transformation from Pt_1_Ag_28_(S-PhMe_2_)_*x*_(S-Adm)_18−*x*_(PPh_3_)_4_ (*x* = 0–6, **Pt1Ag28-2**) to Pt_1_Ag_24_(S-PhMe_2_)_18_ (**Pt1Ag24**) with a spherical configuration or to Pt_1_Ag_28_(S-Adm)_18_(PPh_3_)_4_ (**Pt1Ag28-1**) with a tetrahedral configuration has been accomplished. In addition, the spherical or tetrahedral configuration of the clusters could be reversibly transformed by re-regulating the proportion of counterions with opposite charges. More significantly, the configuration transformation rate has been meticulously manipulated by regulating the polarization effect of the ions on the parent nanoclusters. The observations in this paper provide an intriguing nanomodel that enables the polarization effect to be understood at the atomic level.

## Abstract 2

Based on the inter-conversion between Pt_1_Ag_24_(SR)_18_ and Pt_1_Ag_28_(SR)_18_(PPh_3_)_4_, an insight into the polarization effect in controlling the morphology of metal nanoparticles is presented.

## Graphical abstract

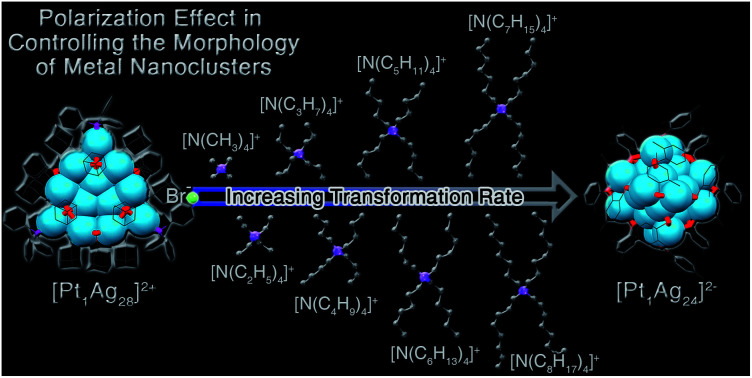

## 1. Introduction

Metal nanoparticles with different morphologies, such as nanostars, nanorods, nanowires, nanoflowers, and so on, have all been the subjects of widespread research interest in the past few decades.^1^ The underlying chemistry is a significant influence on the morphologies of nanoparticles, and on their physicochemical properties, including electrochemical, catalytic, and optical properties.^1^ Several reaction factors (*e.g.*, temperature, stirring speed, reactant, counterions and so on) have been proved to have the capability to control the morphology of nanoparticles.^1,2^ Amongst these factors, the role of counterions (*e.g.*, halides) in controlling the shape of the nanoparticles has become a subject of particular interest.^2*b*–*d*^ However, a detailed understanding of how potential counterion–metal interactions influence the generation of corresponding nanoparticles with different morphologies has remained elusive for two main reasons: (i) the reaction process is hard to track, and (ii) the surface chemistry (*e.g.*, metal–ligand interactions) of the nanoparticles is difficult to study at the atomic level.^3*a*^ These uncertainties impede the deep understanding of the nanoparticle formation as well as the development of the shape control of nanoparticles. Atomic-level understanding of the counterion effect requires more precise molecular entities as model nanosystems and precise molecular tools. For this reason, metal nanoclusters benefit from their monodisperse sizes and accurately characterized structures, and provide an ideal platform to investigate the counterion–nanoparticle interactions at the atomic level.^3–10^

Previous research has come close to a unified conclusion – the control of the introduced salts (*i.e.*, CTAB or CTAC) in the preparation of the nanoparticles is able to control their morphologies.^2^ Mirkin and co-workers have demonstrated that manipulating (i) the ratio of metal to halide ion, and (ii) the selection of appropriate halide ions could rationally control the morphology of the nanoparticles, under otherwise identical preparation conditions.^2*d*^ In this context, it is acceptable that the nature of the counterions plays a crucial role in the growth processes of nanoparticles, and the polarization effect of ion-to-nanoparticle is among one of the most effective in shape control. Nevertheless, several fundamental questions remain largely unexplored: what potential counterion–metal interactions are primarily responsible for the shape control of the nanoparticles? Do the counterions mainly affect the dispersed metals in the growth processes, or just have an effect on the nanoparticles? Could the morphology of the corresponding nanoparticles be manipulated at the atomic level by regulating the species and the amount of counterions added? The counterion–nanoparticle interactions should be comprehended in the nanosystem of established principles of chemistry. In this context, the counterion–nanoparticle interactions as well as the polarization effects of the counterions are to be investigated by using atomically precise nanoclusters with different configurations. This would create a new opportunity for understanding the underlying chemistry of the shape control in nanoparticles.

In this work, the polarization effect in controlling the morphology of nanoclusters was investigated at the atomic level using [Pt_1_Ag_24_(S-PhMe_2_)_18_]^2−^ (**Pt1Ag24**) and [Pt_1_Ag_28_(S-Adm)_18_(PPh_3_)_4_]^2+^ (**Pt1Ag28-1**, S-Adm = adamantanethiol) nanoclusters as templates. The [Pt_1_Ag_28_(S-PhMe_2_)_*x*_(S-Adm)_18−*x*_(PPh_3_)_4_]^2+^ (**Pt1Ag28-2**, *x* = 0–6) nanoclusters could be controllably transformed into **Pt1Ag24** with a spherical configurational or **Pt1Ag28-1** with a tetrahedral configuration by introducing different salts (PPh_4_Br or NaBPh_4_). In addition, by regulating the proportion of the opposite salts (*i.e.*, PPh_4_Br *versus* NaBPh_4_), the spherical or tetrahedral morphology of the cluster shaped products could be reversibly converted, forming a cyclic transformation system. More significantly, the rate of the conversion from the tetrahedral **Pt1Ag28-2** to the spherical **Pt1Ag24** is directly proportional to the magnitude of the polarization effect of ion-to-nanocluster, which could be meticulously manipulated by regulating the interaction distance between opposite-ions and corresponding nanoclusters (*i.e.*, using different sizes of cations in [N(C_*m*_H_2*m*+1_)_4_]^+^Br^−^, *m* = 1–8).

## 2. Experimental methods

### Materials

All reagents were purchased from Sigma-Aldrich and used without further purification: hexachloroplatinic(iv) acid (H_2_PtCl_6_·6H_2_O, 99.99%, metal basis), silver nitrate (AgNO_3_, 99%, metals basis), 2,4-dimethylbenzenethiol (PhMe_2_-SH, 99%), triphenylphosphine (PPh_3_, 99%), 1-adamantanethiol (AdmSH, C_10_H_15_-SH, 99%), sodium borohydride (NaBH_4_, 95%), sodium tetraphenylborate (NaBPh_4_, 98%), tetraphenylphosphonium bromide (PPh_4_Br, 98%), tetramethylphosphonium bromide (PMe_4_Br, 98%), tetraphenylphosphonium chloride (PPh_4_Cl, 98%), tetra-*n*-octylammonium bromide ([N(C_8_H_17_)_4_]Br, TOAB, 98%), tetra-*n*-heptylammonium bromide ([N(C_7_H_15_)_4_]Br, 98%), tetra-*n*-hexylammonium bromide ([N(C_6_H_13_)_4_]Br, 98%), tetra-*n*-amylammonium bromide ([N(C_5_H_11_)_4_]Br, 98%), tetra-*n*-butylammonium bromide ([N(C_4_H_9_)_4_]Br, 98%), tetra-*n*-propylammonium bromide ([N(C_3_H_7_)_4_]Br, 98%), tetraethylammonium bromide ([N(C_2_H_5_)_4_]Br, 98%), tetramethylammonium bromide ([N(CH_3_)_4_]Br, 98%), hydrobromic acid (HBr, 47.0–49.0%), methylene chloride (CH_2_Cl_2_, HPLC grade), methanol (CH_3_OH, HPLC), ethanol (CH_3_CH_2_OH, HPLC).

### Preparation of [PPh_4_]^+^[BPh_4_]^−^

To 10 mL of CH_3_CH_2_OH, 1 mmol of Na^+^[BPh_4_]^−^ and 1 mmol of [PPh_4_]^+^Br^−^ ions were added. After 10 min, the precipitate was collected and further dissolved in CH_2_Cl_2_, giving a solution of [PPh_4_]^+^[BPh_4_]^−^. The preparations of [N(C_*m*_H_2*m*+1_)_4_]^+^[BPh_4_]^−^ (*m* = 4–8) were the same as the synthetic procedure of [PPh_4_]^+^[BPh_4_]^−^, except that the [PPh_4_]^+^[Br]^−^ was altered to [N(C_*m*_H_2*m*+1_)_4_]^+^Br^−^ (*m* = 4–8).

### Synthesis of the [Pt_1_Ag_24_(SPhMe_2_)_18_](PPh_4_)_2_ nanocluster (**Pt1Ag24**)

The preparation of [Pt_1_Ag_24_(SPhMe_2_)_18_](PPh_4_)_2_ was based on a previously reported method.^11^

### Synthesis of the [Pt_1_Ag_28_(S-Adm)_18_(PPh_3_)_4_]Cl_2_ nanocluster (**Pt1Ag28-1**)

The preparation of [Pt_1_Ag_28_(S-Adm)_18_(PPh_3_)_4_]Cl_2_ was based on a previously reported method.^12^

### Synthesis of the [Pt_1_Ag_28_(S-PhMe_2_)_*x*_(S-Adm)_18−*x*_(PPh_3_)_4_]Cl_2_ nanoclusters (**Pt1Ag28-2**)

For the nanocluster synthesis, 20 mg of [Pt_1_Ag_28_(S-Adm)_18_(PPh_3_)_4_]Cl_2_ was dissolved in 10 mL of CH_2_Cl_2_, to which 10 μL of PhMe_2_-SH was added. The reaction was allowed to proceed for 30 min at room temperature. Then, the [Pt_1_Ag_28_(S-PhMe_2_)_*x*_(S-Adm)_18−*x*_(PPh_3_)_4_]Cl_2_ nanoclusters were obtained. The ESI-MS and UV-vis measurements were used to track the ligand-exchange process.

### Conversion from **Pt1Ag28-2** to **Pt1Ag28-1**

Typically, 10 mg of NaBPh_4_ (in 3 mL of CH_2_Cl_2_) was added to the previously mentioned CH_2_Cl_2_ solution of [Pt_1_Ag_28_(S-PhMe_2_)_*x*_(S-Adm)_18−*x*_(PPh_3_)_4_]Cl_2_. The color of the solution slowly altered from yellow to orange. The [Pt_1_Ag_28_(S-Adm)_18_(PPh_3_)_4_](BPh_4_)_2_ nanocluster was generated after 5 min, which was validated by the ESI-MS results.

### Conversion from **Pt1Ag28-2** to **Pt1Ag24**

Typically, 10 mg of PPh_4_Br (in 3 mL of CH_2_Cl_2_) was added to the previously mentioned CH_2_Cl_2_ solution of [Pt_1_Ag_28_(S-PhMe_2_)_*x*_(S-Adm)_18−*x*_(PPh_3_)_4_]Cl_2_. The color of the solution altered from yellow to green instantaneously. The [Pt_1_Ag_24_(SPhMe_2_)_18_](PPh_4_)_2_ nanocluster was generated in several seconds, which was validated by the ESI-MS results.

### Conversion from **Pt1Ag28-1** to **Pt1Ag24**

Typically, 20 mg of [Pt_1_Ag_28_(S-Adm)_18_(PPh_3_)_4_]Cl_2_ was dissolved in 10 mL of CH_2_Cl_2_. Then 10 mg of PPh_4_Br (in 3 mL of CH_2_Cl_2_) and 200 μL of PhMe_2_-SH were added simultaneously to the solution. The color of the solution altered from orange to green instantaneously, demonstrating the fast generation of [Pt_1_Ag_24_(SPhMe_2_)_18_](PPh_4_)_2_, which was further validated by the ESI-MS results.

### Cyclic conversion between **Pt1Ag28-1** and **Pt1Ag24**

To the [Pt_1_Ag_28_(S-Adm)_18_(PPh_3_)_4_](BPh_4_)_2_ solution (obtained from the aforementioned conversion from **Pt1Ag28-2** to **Pt1Ag28-1**), 25 mg of PPh_4_Br (twice the mole ratio of NaBPh_4_) was added. The color of the solution altered from orange to green instantaneously, demonstrating the fast generation of [Pt_1_Ag_24_(SPhMe_2_)_18_](PPh_4_)_2_. Then, to this solution, 30 mg of NaBPh_4_ (*n* + 1 times the mole ratio of the initial NaBPh_4_, where “*n*” represents the cycle times) was added. The color gradually altered from green to orange (quite slow compared with the generation of [Pt_1_Ag_24_(S-PhMe_2_)_18_](PPh_4_)_2_), demonstrating the slow generation of [Pt_1_Ag_28_(S-Adm)_18_(PPh_3_)_4_](BPh_4_)_2_. All of these processes were tracked by UV-vis and ESI-MS measurements.

### Conversion from **Pt1Ag28-2** to [Pt_1_Ag_24_(SPhMe_2_)_18_][N(C_*m*_H_2*m*+1_)]_2_ (*m* = 1–8)

Typically, 10 mg of [N(C_*m*_H_2*m*+1_)]Br (*m* = 1–8) was added to the previously mentioned CH_2_Cl_2_ solution of [Pt_1_Ag_28_(S-PhMe_2_)_*x*_(S-Adm)_18−*x*_(PPh_3_)_4_]Cl_2_. The color of the solution altered from yellow to green, and the [Pt_1_Ag_24_(S-PhMe_2_)_18_][N(C_*m*_H_2*m*+1_)]_2_ nanoclusters were generated. The conversions were performed at −37 °C as this slowed down the reaction. The UV-vis measurement was performed to track the conversion, and to determine the generation rate of the [Pt_1_Ag_24_(S-PhMe_2_)_18_][N(C_*m*_H_2*m*+1_)]_2_ nanoclusters.

### Characterizations

All the UV-vis absorption spectra of the nanoclusters dissolved in CH_2_Cl_2_ were recorded using an Agilent 8453 diode array spectrometer, and the background correction was made using a CH_2_Cl_2_ blank.

Electrospray ionization time-of-flight mass spectrometry (ESI-TOF-MS) measurement was performed on a Bruker Daltonics MicrOTOF-Q III high-resolution mass spectrometer. The sample was directly infused into the chamber at 5 μL min^−1^. To prepare the ESI, sample, the nanoclusters were dissolved in CH_2_Cl_2_ (1 mg mL^−1^) and diluted (v/v = 1 : 2) with dry methanol.

## 3. Results and discussion

**Fig. 1:**
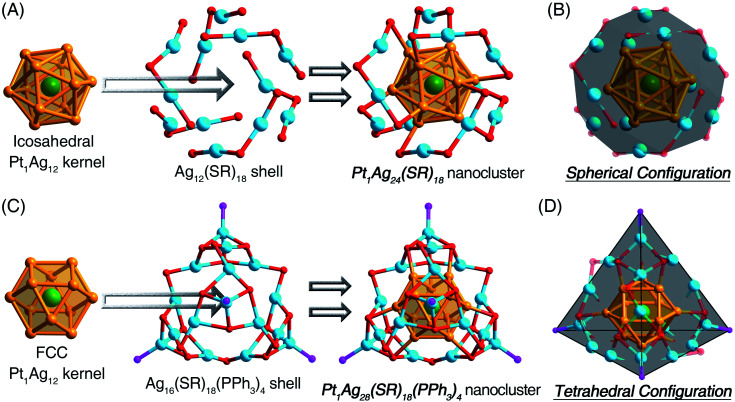
(A) Structural anatomy of **Pt1Ag24**. (B) Illustration of the spherical configuration of **Pt1Ag24**. (C) Structural anatomy of **Pt1Ag28-1**. (D) Illustration of the tetrahedral configuration of **Pt1Ag28-1**. Color legends: dark green sphere: Pt, orange sphere: Ag in the kernel, light blue sphere: Ag on the shell, red sphere: S, purple sphere: P. For clarity, all the C atoms and the H atoms are omitted.

For a better understanding of the configurations of the nanoclusters that are discussed in this work, their structural anatomies are shown in Fig. 1 (and see also Fig. S1 and S2, ESI† for the overall structures). The **Pt1Ag24** nanocluster contains an icosahedral Pt_1_Ag_12_ kernel, which is further stabilized by an Ag_12_(S-PhMe_2_)_18_ shell (Fig. 1A).^11^ For comparison, the **Pt1Ag28-1** nanocluster comprises an fcc Pt_1_Ag_12_ kernel and an Ag_16_(S-Adm)_18_(PPh_3_)_4_ shell (Fig. 1C).^12^ Although the crystal structure of **Pt1Ag28-2** was not obtained, **Pt1Ag28-2** should exhibit a comparable structure to **Pt1Ag28-1** because of they have the same metal–ligand compositions and similar optical absorptions. In this context, the overall structure of **Pt1Ag24** displayed a spherical configuration (Fig. 1B), whereas both **Pt1Ag28-1** and **Pt1Ag28-2** followed a tetrahedral configuration (Fig. 1D and S3, ESI†).^11,12^

**Fig. 2:**
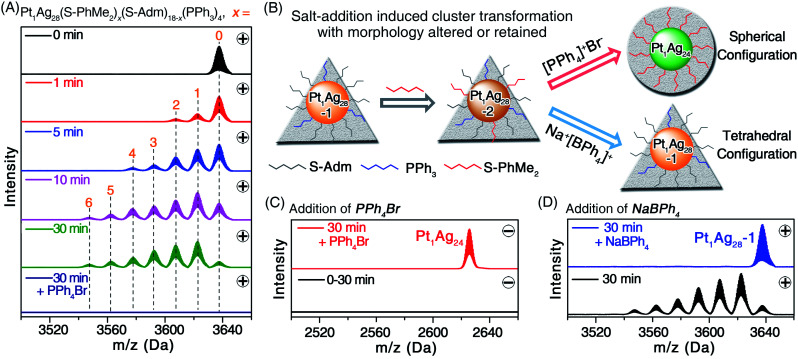
(A and C) Time-dependent ESI-MS spectra of the ligand-exchange process from **Pt1Ag28-1** to **Pt1Ag28-2** (0–30 min) and the PPh_4_Br addition induced morphology change process from **Pt1Ag28-2** to **Pt1Ag24** (30 min + PPh_4_Br). (A) Is detected in the positive mode, and (C) is detected in the negative mode. (B) Illustration of the ligand-exchange process and the morphology control processes. The triangular background represents the tetrahedral configuration of **Pt1Ag28-1** and **Pt1Ag28-2**, and the circular background represents the spherical configuration of **Pt1Ag24**. (D) The ESI-MS results of the NaBPh_4_ addition induced conversion from **Pt1Ag28-2** to **Pt1Ag28-1**, detected in the positive mode.

The **Pt1Ag28-2** was prepared *via* a ligand exchange with the **Pt1Ag28-1** with HS-PhMe_2_ ligands (Fig. 2A), in which process the tetrahedral configuration of **Pt1Ag28** was retained (Fig. 2B and S3, ESI†). As demonstrated in Fig. 2A, the substitution of S-Adm by S-PhMe_2_ on the surface of **Pt1Ag28-1** was processed ligand by ligand (see Fig. S4, ESI† for the expansion of the ESI-MS results). Finally, the maximum ratio of S-PhMe_2_/S-Adm was 1/2, *i.e.*, [Pt_1_Ag_28_(S-PhMe_2_)_6_(S-Adm)_12_(PPh_3_)_4_]^2+^ (Fig. 2A). No nanocluster with a negative charge accompanied by the ligand-exchange process was detected (Fig. 2A, dark blue line).

To the previously mentioned solution of **Pt1Ag28-2**, PPh_4_Br was added, which triggered the transformation from the tetrahedral **Pt1Ag28-2** to the spherical **Pt1Ag24**, as shown by ESI-MS results (Fig. 2A–C). Considering that the introduction of PPh_4_Br was a unique variable, it is safe to say that the ion–nanocluster interactions induced the complete substitution of the PPh_3_ and S-Adm ligands with S-PhMe_2_ and this further activated the morphology change process (Fig. 2B and S5A, ESI†). In contrast, the final product would be **Pt1Ag28-1** when the introduced PPh_4_Br was replaced by adding the NaBPh_4_ to the solution of **Pt1Ag28-2**, throughout which process the configuration of nanoclusters was maintained as a tetrahedron (Fig. 2B, D, and S5B, ESI†). Significantly, the control over the nanocluster morphologies as tetrahedral or spherical configurations was accomplished together with these ion-addition processes.

The time-dependent UV-vis spectra of the transformations from **Pt1Ag28-1** to **Pt1Ag28-2** and from **Pt1Ag28-2** to **Pt1Ag28-1** or **Pt1Ag24** were then tracked (Fig. S6, ESI†). The main absorptions (445 nm) of **Pt1Ag28-1** and **Pt1Ag28-2** nanoclusters were the same, whereas the UV-vis spectrum of **Pt1Ag28-2** exhibited a scarcely noticeable shoulder band at about 530 nm (Fig. S6A and B, ESI†). Firstly, the similar optical absorptions between **Pt1Ag28-1** and **Pt1Ag28-2** demonstrated their comparable electronic/geometric structures.^12*c*^ In addition, the slight difference in optical absorptions might give rise to the color diversity between the CH_2_Cl_2_ solutions of these two nanoclusters (*i.e.*, the orange color of **Pt1Ag28-1** and the yellow color of **Pt1Ag28-2**). Also, some nanocluster entities might be decomposed by the ligand-exchange process from **Pt1Ag28-1** to **Pt1Ag28-2**, which also resulted in the color of the reaction solution lightening.

For the transformation from **Pt1Ag28-2** to **Pt1Ag24**, the rapid change in optical absorptions further demonstrated its fast conversion rate (Fig. S6C, ESI†). In addition, such a conversion proceeded with a high yield (>80%; Fig. S6C, ESI†). Considering that the **Pt1Ag28** nanocluster framework contained several PPh_3_-containing surface structures that were absent in **Pt1Ag24**, the ESI-MS of its raw solution was determined (Fig. 2A, dark blue line, the sample of “30 min + PPh_4_Br”) to track these PPh_3_-containing units. As shown in Fig. S7, ESI†, several mass signals of PPh_3_-containing complexes were observed. However, no nanocluster intermediate was detected, and this was probably because of the rapid transformation which meant that the intermediates were hard to detect, or the possible intermediates were unstable that they would spontaneously transform into **Pt1Ag28** or **Pt1Ag24** nanoclusters.

**Fig. 3:**
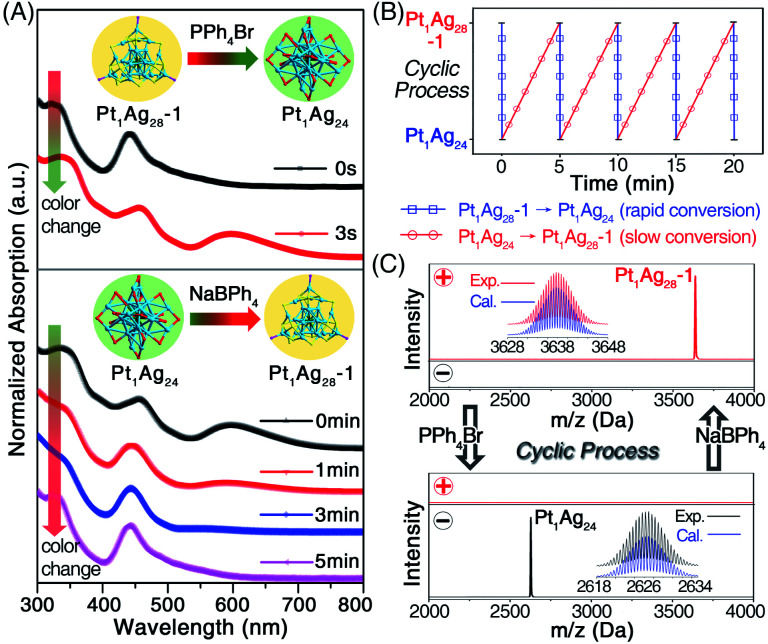
(A) The UV-vis spectra of the cyclic reaction between **Pt1Ag24** and **Pt1Ag28-1** induced by the addition of PPh_4_Br or NaBPh_4_. The arrows represent the color changes accompanied by the cyclic processes. (B) The cyclic transformation between the **Pt1Ag24** and **Pt1Ag28-1** nanoclusters. (C) The ESI-MS tracking of the cyclic reaction in the positive- or negative-mode.

The previously mentioned results illustrated the generation of diverse cluster products with different morphologies (*i.e.*, sphere or tetrahedron) induced by the addition of PPh_4_Br or NaBPh_4_. By noticing that the **Pt1Ag24** and **Pt1Ag28-1** could be obtained from the same cluster intermediate (*i.e.*, **Pt1Ag28-2**), it was perceived as a good opportunity to achieve the inter-conversion between these two nanoclusters with different configurations. As shown in Fig. 3A, to the CH_2_Cl_2_ solution of **Pt1Ag28-1** (raw solution without any purification that contained HS-PhMe_2_, HS-Adm, and PPh_3_ ligands, see the Experimental methods), the addition of an excess amount of PPh_4_Br induced the fast conversion from **Pt1Ag28-1** to **Pt1Ag24** within 3 s, during which process the solution turned from orange to green. It should be noted that the conversion from **Pt1Ag28-1** to **Pt1Ag24** should go *via***Pt1Ag28-2**, which was hard to detect due to the rapid conversion (*i.e.*, within 3 s). Conversely, a further excessive dose of the NaBPh_4_ resulted in the re-generation of **Pt1Ag28-1** from **Pt1Ag24**, however, this conversion was quite a lot slower relative to its opposite process (Fig. 3A). Collectively, a cyclic transformation between **Pt1Ag24** and **Pt1Ag28-1** nanoclusters was achieved (Fig. 3B), during which processes the control over the nanocluster morphologies between spherical and tetrahedral configurations was fulfilled. The ESI-MS measurements were performed to validate the cycle process (Fig. 3C), and the retained mass results until the fourth cycle suggested the steadiness of the cyclic system. It should be noted that the conversion from **Pt1Ag28-1** to **Pt1Ag24** was much more rapid than the reverse process (within 3 s *versus* 5 min, as shown in Fig. 3B). Two possible reasons were proposed to explain the remarkably different conversion rates: (i) the relatively small amount of HS-Adm compared to HS-PhMe_2_,^13^ and (ii) the much more favorable conversion from **Pt1Ag28-1** to **Pt1Ag24** relative to the reverse process. Indeed, the ejection of four Ag atoms (intra-cluster behavior) during the conversion from **Pt1Ag28-1** to **Pt1Ag24** was anticipated to be easier than the reverse process wherein the extraction of four Ag atoms is included (this might be an inter-cluster behavior).

Previous research has demonstrated the crucial role of ions (or salts) in the preparation of metal nanoclusters.^14^ For example, in the synthesis of the [Au_25_(S-PhC_2_H_4_)_18_]^−^ nanocluster, the TOAB was not only exploited as a phase transfer agent, but also acted as a counterion (TOA^+^) for balancing the “−1” charge of Au_25_(S-PhC_2_H_4_)_18_. In addition, the presence of [PPh_4_]^+^Br^−^ contributed to the high yield for the syntheses of [Ag_25_(SR)_18_]^−^, [Ag_44_(SR)_30_]^4−^, and so. Most previous research has focused on the counterion part of the introduced salts (*e.g.*, [TOA]^+^ or [PPh_4_]^+^), however, the effect of the remaining ions (*e.g.*, Br^−^ or Cl^−^) received little interest. In other words, it remains unexplored as to whether the [cation]^+^[anion]^−^ takes effect as a whole on the cluster synthesis. With regard to this work, a fundamental but significant question arose: what is the underlying chemistry of ion addition-induced nanocluster transformation?

**Scheme 1:**
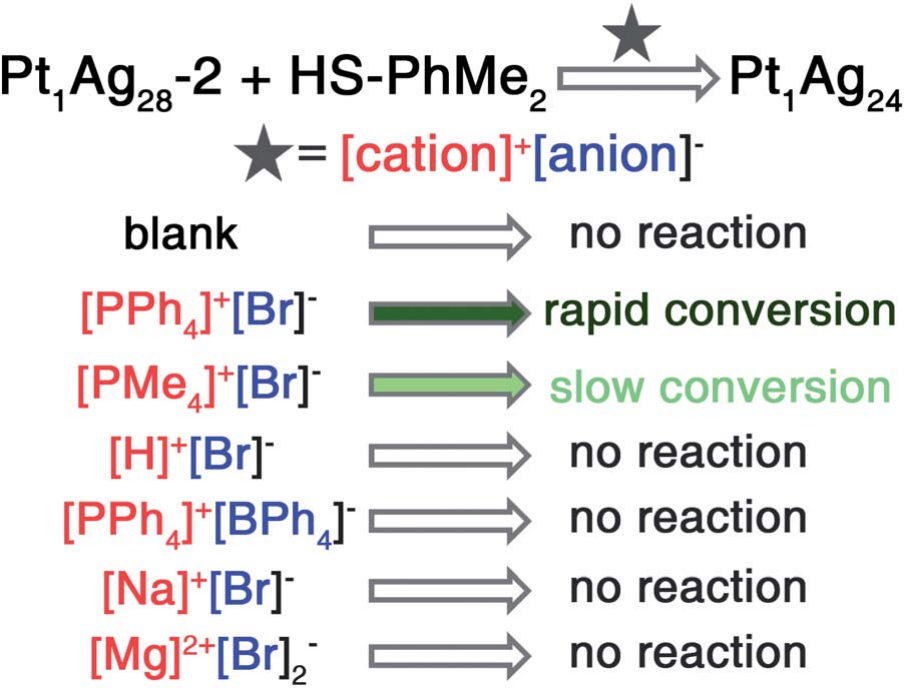
Transformation rates from **Pt1Ag28-2** to **Pt1Ag24** induced by the addition of different salts.

Here, the control over the introduced salts was performed by transforming the tetrahedral **Pt1Ag28-2** into the spherical **Pt1Ag24**. It should be noted that the precise structures of both **Pt1Ag28-2** and **Pt1Ag24** nanoclusters rendered them ideal nanomodels for the atomic-level analysis of the ion-induced transformation. As shown in Scheme 1, the transformation from **Pt1Ag28-2** to **Pt1Ag24** was activated by different [cation]^+^[anion]^−^ such as [PPh_4_]^+^Br^−^, [PMe_4_]^+^Br^−^, H^+^Br^−^, or [PPh_4_]^+^[BPh_4_]^−^. For the CH_2_Cl_2_ solution of **Pt1Ag28-2**, although the addition of [PPh_4_]^+^Br^−^ or [PMe_4_]^+^Br^−^ could both trigger the transformation from **Pt1Ag28-2** to **Pt1Ag24**, with [PMe_4_]^+^Br^−^ the transformation was much slower (10 s *versus* 3 s for the color change from orange to green). Such a noticeable slowness resulted from the size disparity between the [PPh_4_]^+^ and [PMe_4_]^+^ cations. In addition, the addition of H^+^Br^−^, [PPh_4_]^+^[BPh_4_]^−^, Na^+^Br^−^, or Mg^2+^Br_2_^−^ had no impact on the cluster system and the cluster remained as **Pt1Ag28-2** (Scheme 1), which eliminated the possibility that [PPh_4_]^+^/[PMe_4_]^+^ or Br^−^ could solely cause the transformation. In other words, the cations (*i.e.*, [PPh_4_]^+^) and anions (*i.e.*, Br^−^) worked together to activate the cluster transformation.

**Fig. 4:**
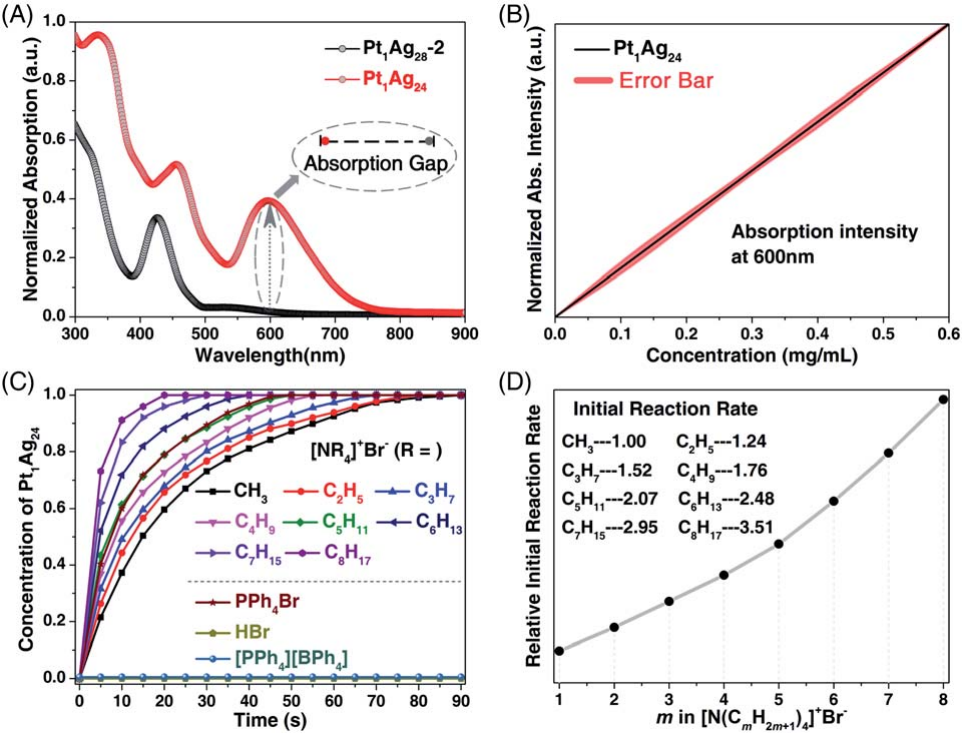
(A) Comparison between the UV-vis spectra between **Pt1Ag28-2** and **Pt1Ag24** and the absorption gap at 600 nm. (B) The **Pt1Ag24** concentration-dependent absorptions at 600 nm. (C) Time-dependent concentration of the prepared **Pt1Ag24** induced by the addition of [N(C_*m*_H_2*m*+1_)_4_]^+^Br^−^ (*m* = 1–8). (D) Comparison of the initial reaction rates in these transformations.

For a deep understanding of the ion-induced transformation from **Pt1Ag28-2** to **Pt1Ag24**, the reason why the transformation rates show a remarkable difference when induced by [PPh_4_]^+^Br^−^ or [PMe_4_]^+^Br^−^ should be clear. In this context, the [N(C_*m*_H_2*m*+1_)_4_]^+^Br^−^ (*m* = 1–8) with gradually growing cations and an unchanged anion were further used to activate the transformation. Considering the apparent enhancement of the UV-vis absorption at 600 nm from **Pt1Ag28-2** (with almost no absorption) to **Pt1Ag24** (with strong absorption), the optical absorption intensity at 600 nm was monitored to characterize the generation of **Pt1Ag24** (Fig. 4A). Indeed, the concentration of **Pt1Ag24** in solution was determined to be directly proportional to the absorption intensity at 600 nm, which was in agreement with the Beer–Lambert law (Fig. 4B).^15^

Considering that the transformation from the tetrahedral **Pt1Ag28-2** to the spherical **Pt1Ag24** was too fast to be monitored, the time-dependent UV-vis spectra were determined at −37 °C to slow down the reaction rate. Fig. S8 (ESI†) shows the time-dependent UV-vis evolutions from **Pt1Ag28-2** to **Pt1Ag24** induced by the addition of [N(C_*m*_H_2*m*+1_)_4_]^+^Br^−^ (*m* = 1–8). According to the absorptions measured at 600 nm, the time-dependent concentrations of **Pt1Ag24** in the solution were obtained. As shown in Fig. 4C, accompanied by the addition of [N(C_*m*_H_2*m*+1_)_4_]^+^Br^−^, the **Pt1Ag24** nanocluster was generated rapidly in the beginning, and then the generation rate leveled off, and finally, all the **Pt1Ag28-2** was converted into the **Pt1Ag24**. In addition, compared with the rapid reaction with [N(C_8_H_17_)_4_]^+^Br^−^ (within 20 s), the reaction of [N(CH_3_)_4_]Br was relatively slow and was completed after 90 s (Fig. 4C and S8, ESI†). In fact, the overall reaction rate was proportional to the length of the carbon chain in the cations (or its steric hindrance) of [N(C_*m*_H_2*m*+1_)_4_]^+^Br^−^ (Fig. 4C). To simplify the process, the initial rate of these conversions was compared – the initial rate of [N(C_8_H_17_)_4_]^+^Br^−^ was 3.51 when the initial ratio of [N(CH_3_)_4_]^+^Br^−^ was set as 1.00, and the initial rates were also proportional to the C_*m*_H_2*m*+1_ lengths in the corresponding [N(C_*m*_H_2*m*+1_)_4_]^+^Br^−^ (Fig. 4D).^16^

Having obtained these ion addition-induced conversions from the tetrahedral **Pt1Ag28-2** to the spherical **Pt1Ag24**, it was proposed that the underlying chemistry was the polarization effect of the ions introduced (inducing both cations and anions) to the nanoclusters. The explanation for this is given next.

**Scheme 2:**
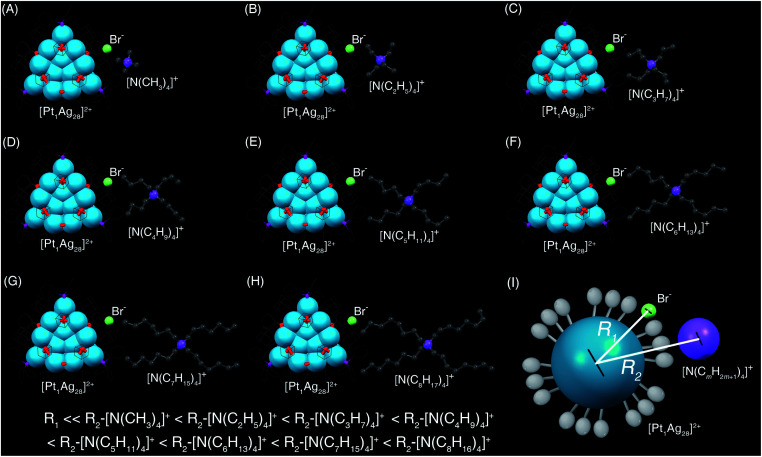
Transformation from **Pt1Ag28-2** to **Pt1Ag24** induced by the addition of different salts.

The introduced [N(C_*m*_H_2*m*+1_)_4_]^+^Br^−^ was separated into two parts: the larger [N(C_*m*_H_2*m*+1_)_4_]^+^ cation and the smaller Br^−^ anion (Scheme 2). Because of the steric effect and the charge effect, the distances between the **Pt1Ag28** cluster framework and the cations or the anions were different. Specifically, the large steric hindrance between the peripheral ligands in **Pt1Ag28** and the [N(C_*m*_H_2*m*+1_)_4_]^+^ hindered these cations to approach the cluster kernel. Conversely, the Br^−^ anion with a small size kept close to the cluster kernel. In addition, the **Pt1Ag28** and the Br^−^ were attractive because of their opposite charges (“+2” *versus* “−1”) whereas the **Pt1Ag28** cluster and the [N(C_*m*_H_2*m*+1_)_4_]^+^ cation were repulsive because of they had the same charges (“+2” *versus* “+1”). In this context, the Br^−^ anion should be closer to the nanocluster relative to its corresponding cation (Scheme 2), and then, the polarization effect arising from the [cation]^+^[anion]^−^ pair further acted on the cluster and induced the framework transformation from **Pt1Ag28** into **Pt1Ag24**.

For the different conversion rates in the corresponding [N(C_*m*_H_2*m*+1_)_4_]^+^Br^−^ addition-induced transformations, although these [N(C_*m*_H_2*m*+1_)_4_]^+^ cations displayed the same “+1” valence state, they moved away from the cluster as *m* grew from one to eight because of their increasing steric hindrance to the cluster. However, the distance between the Br^−^ anion and the cluster remained unchanged (Scheme 2). Accordingly, the polarization effect of Br^−^ to the cluster kernel (or the interaction between Br^−^ and the cluster) was intensified as *m* grew, which further accelerated the cluster transformation (Scheme 2). Such an explanation was also applicable to the different conversion rates of [PPh_4_]^+^Br^−^ or [PMe_4_]^+^Br^−^ (Scheme 1). It should be noted that an attempt to convert the **Pt1Ag28-1** nanocluster was made by introducing PPh_4_Br without ligand exchange. However, the nanocluster remained as **Pt1Ag28-1**, and did not transform into Pt_1_Ag_24_(S-Adm)_18_, demonstrating that the nanocluster transformation resulted from the proposed polarization effect, but not from the ligand effect.

Then, the Br^−^ anion in [N(C_*m*_H_2*m*+1_)_4_]^+^Br^−^ was altered into the [BPh_4_]^−^ (*i.e.*, [N(C_*m*_H_2*m*+1_)_4_]^+^[BPh_4_]^−^, *m* = 4–8) for further verification. As shown in Fig. S9 (ESI†), the addition of [N(C_6_H_13_)_4_]^+^[BPh_4_]^−^, [N(C_7_H_15_)_4_]^+^[BPh_4_]^−^, or [N(C_8_H_17_)_4_]^+^[BPh_4_]^−^ to the solution of **Pt1Ag28-2** can activate the cluster transformation, but not for the [N(C_4_H_9_)_4_]^+^[BPh_4_]^−^ or [N(C_5_H_11_)_4_]^+^[BPh_4_]^−^. It should be noted that the steric hindrances of [N(C_5_H_11_)_4_]^+^ and [BPh_4_]^−^ were almost the same (Scheme 1 and Fig. 4C, brown and green lines), and thus the [N(C_5_H_11_)_4_]^+^[BPh_4_]^−^ should have counterbalanced the polarization effect to the nanocluster. By comparison, the size (or the steric hindrance) of the cation was larger than that of the corresponding anion (*i.e.*, [BPh_4_]^−^) for [N(C_6_H_13_)_4_]^+^[BPh_4_]^−^, [N(C_7_H_15_)_4_]^+^[BPh_4_]^−^, and [N(C_8_H_17_)_4_]^+^[BPh_4_]^−^, and thus the induced polarization effect activated the cluster transformation. Indeed, the transformation rate was accelerated as the size of the cation increased from [N(C_6_H_13_)_4_]^+^ to [N(C_7_H_15_)_4_]^+^ and [N(C_8_H_17_)_4_]^+^ (Fig. S9, ESI†).

## 4. Conclusions

In summary, based on the inter-transformation between Pt_1_Ag_28_(S-Adm)_18_(PPh_3_)_4_ with a tetrahedral configuration and Pt_1_Ag_24_(S-PhMe_2_)_18_ with a spherical configuration, the detailed polarization effect of ions on the nanoparticles has been investigated at the atomic level. The intermediate product Pt_1_Ag_28_(S-PhMe_2_)_*x*_(S-Adm)_18−*x*_(PPh_3_)_4_ could be controllably transformed into spherical **Pt1Ag24** or tetrahedral **Pt1Ag28** by simply regulating the introduced salts, which further formed a cyclic conversion system. It is significant that the rate of transforming the tetrahedral Pt_1_Ag_28_(S-PhMe_2_)_*x*_(S-Adm)_18−*x*_(PPh_3_)_4_ to the spherical Pt_1_Ag_24_(S-PhMe_2_)_18_ is directly proportional to the polarization magnitude of the ions introduced into the nanoclusters, which was meticulously controlled by regulating the interaction distance between the opposite ions and the corresponding nanoclusters (*i.e.*, using different cations in [N(C_*m*_H_2*m*+1_)_4_]^+^Br^−^).

Indeed, the control over introduced salts (*e.g.*, CTAB or CTAC) has been pursued for several decades in the preparation of nanoparticles, while the underlying chemistry of this control remains elusive at the atomic level. It is hoped that the polarization effect proposed in this work can help to promote the understanding of the ion effect in nanoparticle syntheses, and further guide such syntheses. Overall, this work presents a maneuverable interconversion between two nanoclusters with different configurations, based on which the insights, at the atomic level, into the polarization effect in controlling the morphology of metal nanoparticles are presented. Future efforts will focus on the application of the polarization effect to fabricate more nanoclusters and nanoparticles with customized structures and morphologies.

## Data availability

All the data supporting this article have been included in the main text and the ESI.

## Author contributions

X. K. carried out experiments, analyzed the data and wrote the manuscript. X. W. assisted the UV-*vis* analysis and completed the manuscript. S. W. and M. Z. designed the project, analyzed the data, and revised the manuscript.

## Conflicts of interest

There are no conflicts to declare.

## Supplementary Material

SC-012-D1SC00632K-s001
