# Supplementary material for: An insight, at the atomic level, into the polarization effect in controlling the morphology of metal nanoclusters
Source: Chem Sci. 2021 Jul 13;12(33):11080–8. doi: 10.1039/d1sc00632k (PMC8386652; doi:10.1039/d1sc00632k)
Supplement: SC-012-D1SC00632K-s001 [file SC-012-D1SC00632K-s001.pdf]

Electronic Supplementary Material (ESI) for Chemical Science.
This journal is © The Royal Society of Chemistry 2021

Supporting Information:

Atomic Insight into the Polarization Effect in Controlling the Morphology of Metal Nanoclusters

Xi Kang,[1,2] Xiao Wei,[1,2] Shuxin Wang,[1,2,*] Manzhou Zhu[1,2,*]

[1]Department of Chemistry and Centre for Atomic Engineering of Advanced Materials, Anhui Province Key Laboratory of Chemistry for Inorganic/Organic Hybrid Functionalized Materials, Anhui University, Hefei 230601, P. R. China.
[2]Key Laboratory of Structure and Functional Regulation of Hybrid Materials, Anhui University, Ministry of Education, Hefei 230601, P. R. China.

*E-mails of corresponding authors: ixing@ahu.edu.cn (S.W.); zmz@ahu.edu.cn (M.Z.).

Notes: The authors declare no competing financial interest.

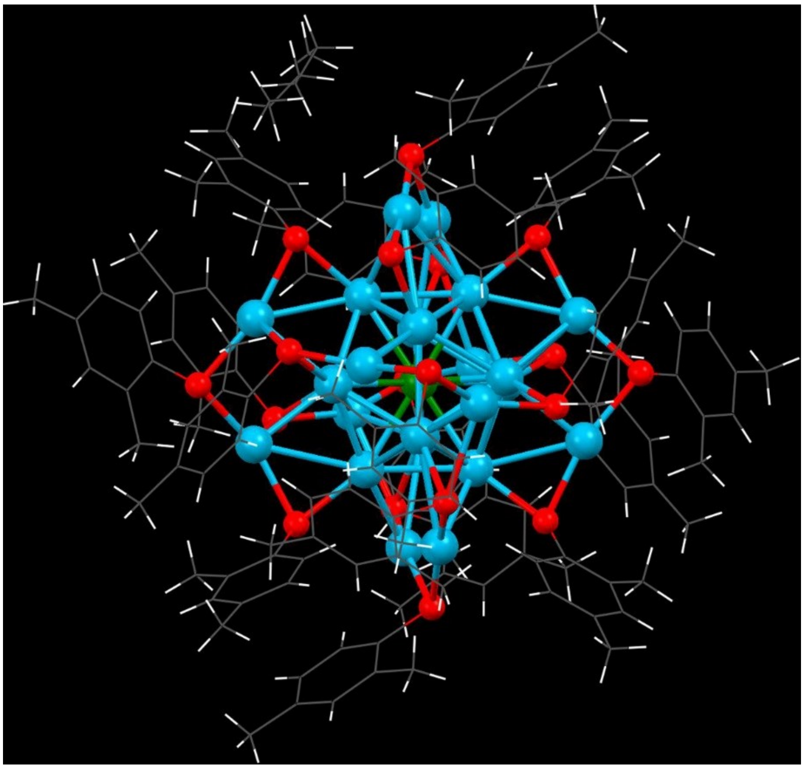

Fig. S1 Total structure of the $[Pt_1Ag_{24}(S\text{-}PhMe_2)_{18}]^{2-}$ nanocluster. Color legends: dark green sphere, Pt; light blue sphere, Ag; red sphere, S; grey sphere, C; white sphere, H.

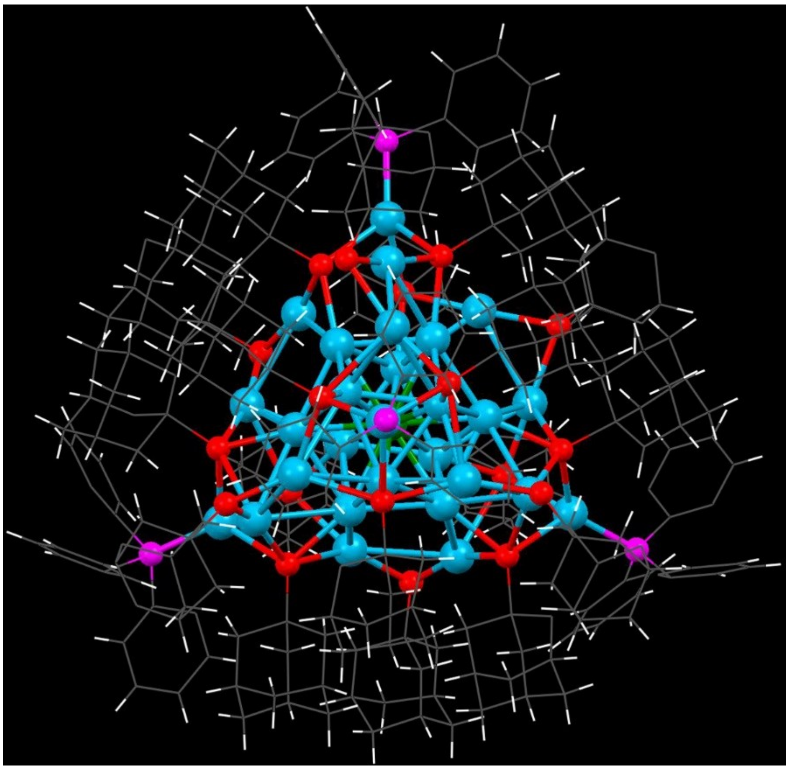

Fig. S2 Total structure of the $[Pt_1Ag_{28}(S\text{-}Adm)_{18}(PPh_3)_4]^{2+}$ nanocluster. Color legends: dark green sphere, Pt; light blue sphere, Ag; red sphere, S; purple sphere, P; grey sphere, C; white sphere, H.

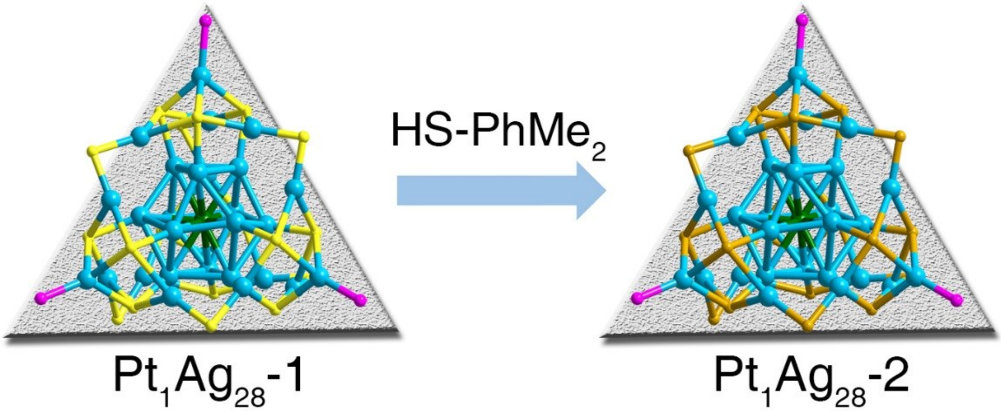

Fig. S3 Illustration of the ligand-exchange process from $[Pt_1Ag_{28}(S\text{-}Adm)_{18}(PPh_3)_4]^{2+}$ to $[Pt_1Ag_{28}(S\text{-}PhMe_2)_x(S\text{-}Adm)_{18\text{-}x}(PPh_3)_4]^{2+}$. Among the ligand-exchange process the tetrahedral configuration of the nanocluster is retained. The triangle background represents the tetrahedral configuration of both $[Pt_1Ag_{28}(S\text{-}Adm)_{18}(PPh_3)_4]^{2+}$ and $[Pt_1Ag_{28}(S\text{-}PhMe_2)_x(S\text{-}Adm)_{18\text{-}x}(PPh_3)_4]^{2+}$ nanoclusters. Color legends: dark green sphere, Pt; light blue sphere, Ag; yellow sphere, S from HS-Adm; dark yellow sphere, S from a mixture of S-PhMe$_2$ and HS-Adm; purple sphere, P. For clarity, all C atoms and H atoms are omitted.

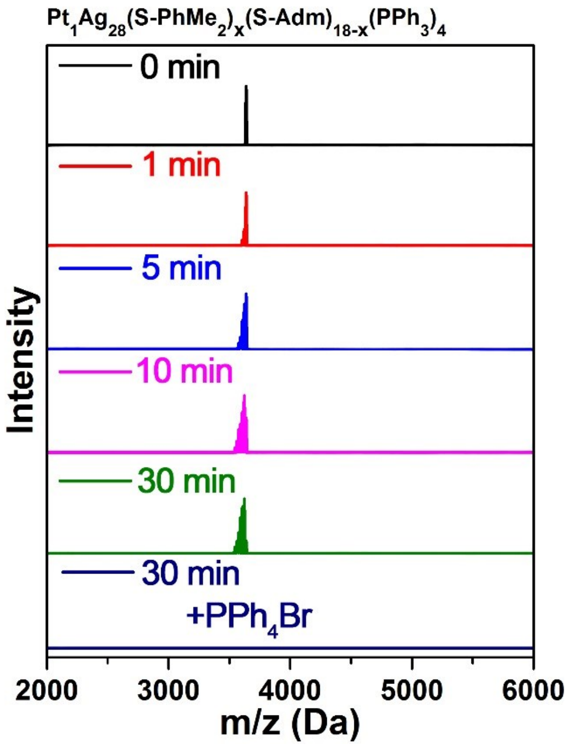

Fig. S4 Time-dependent ESI-MS results of the ligand-exchange reaction between $[Pt_1Ag_{28}(S\text{-}Adm)_{18}(PPh_3)_4]^{2+}$ and $HS\text{-}PhMe_2$ to produce the $[Pt_1Ag_{28}(S\text{-}PhMe_2)_x(S\text{-}Adm)_{18\text{-}x}(PPh_3)_4]^{2+}$ nanoclusters (from black to red, blue, purple, and green). Then, the addition of PPh_4Br induced the transformation from $[Pt_1Ag_{28}(S\text{-}PhMe_2)_x(S\text{-}Adm)_{18\text{-}x}(PPh_3)_4]^{2+}$ to $[Pt_1Ag_{24}(S\text{-}PhMe_2)_{18}]^{2-}$, and thus no signal was observed in the mass spectrum with positive mode (dark blue).

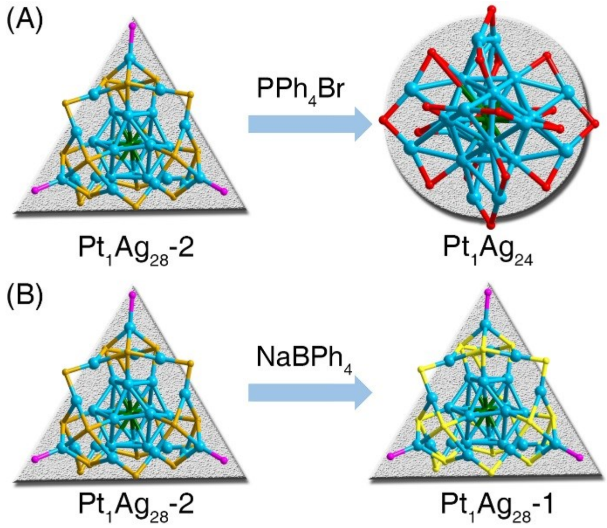

Fig. S5 (A) Illustration of the PPh_4Br addition induced transformation from $[Pt_1Ag_{28}(S\text{-}PhMe_2)_x(S\text{-}Adm)_{18\text{-}x}(PPh_3)_4]^{2+}$ to $Pt_1Ag_{24}(S\text{-}PhMe_2)_{18}$, among which process the configuration of the nanocluster is transformed from tetrahedron to sphere. (B) Illustration of the $NaBPh_4$ addition induced transformation from $[Pt_1Ag_{28}(S\text{-}PhMe_2)_x(S\text{-}Adm)_{18\text{-}x}(PPh_3)_4]^{2+}$ to $[Pt_1Ag_{28}(S\text{-}Adm)_{18}(PPh_3)_4]^{2+}$, among which process the tetrahedral configuration of the nanocluster is retained. Color legends: dark green sphere, Pt; light blue sphere, Ag; yellow sphere, S from S-Adm; red sphere, S from S-PhMe₂; dark yellow sphere, S from a mixture of S-PhMe₂ and S-Adm; purple sphere, P. For clarity, all C atoms and H atoms are omitted.

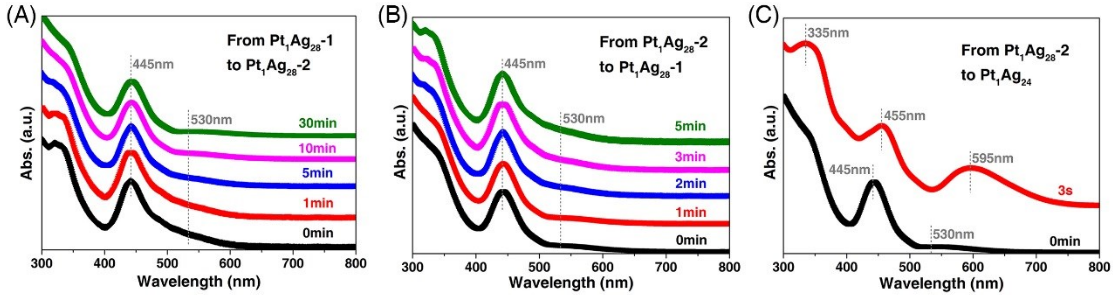

Fig. S6 Time-dependent UV-vis spectra of the transformation (A) from $[Pt_1Ag_{28}(S\text{-}Adm)_{18}(PPh_3)_4]^{2+}$ to $[Pt_1Ag_{28}(S\text{-}PhMe_2)_x(S\text{-}Adm)_{18\text{-}x}(PPh_3)_4]^{2+}$, (B) from $[Pt_1Ag_{28}(S\text{-}PhMe_2)_x(S\text{-}Adm)_{18\text{-}x}(PPh_3)_4]^{2+}$ to $[Pt_1Ag_{28}(S\text{-}Adm)_{18}(PPh_3)_4]^{2+}$, or (C) from $[Pt_1Ag_{28}(S\text{-}PhMe_2)_x(S\text{-}Adm)_{18\text{-}x}(PPh_3)_4]^{2+}$ to $[Pt_1Ag_{24}(S\text{-}PhMe_2)_{18}]^{2-}$. For evaluating the conversion yield from $Pt_1Ag_{28}(S\text{-}PhMe_2)_x(S\text{-}Adm)_{18\text{-}x}(PPh_3)_4$ to $Pt_1Ag_{24}(S\text{-}PhMe_2)_{18}$, 20 mg of $Pt_1Ag_{28}(S\text{-}PhMe_2)_x(S\text{-}Adm)_{18\text{-}x}(PPh_3)_4$ nanoclusters (purified to remove abundant complexes and ligands), 20 µL of $PhMe_2$-SH, and 10 mg of PPh_4Br were added into 20 mL of CH_2Cl_2 under vigorously stirring. The product was washed by MeOH and *n*-hexane several times to produce the pure $Pt_1Ag_{24}(S\text{-}PhMe_2)_{18}$ nanocluster. 13 mg $Pt_1Ag_{24}(S\text{-}PhMe_2)_{18}$ nanocluster was obtained after drying. The molecular weight of $Pt_1Ag_{28}(S\text{-}PhMe_2)_x(S\text{-}Adm)_{18\text{-}x}(PPh_3)_4$ was about 7000 Da, indicating that the conversion yield of the transformation from $Pt_1Ag_{28}(S\text{-}PhMe_2)_x(S\text{-}Adm)_{18\text{-}x}(PPh_3)_4$ to $Pt_1Ag_{24}(S\text{-}PhMe_2)_{18}$ was >80%, i.e., (13mg/5254Da)/(20mg/7000Da) ≈ 86.6%.

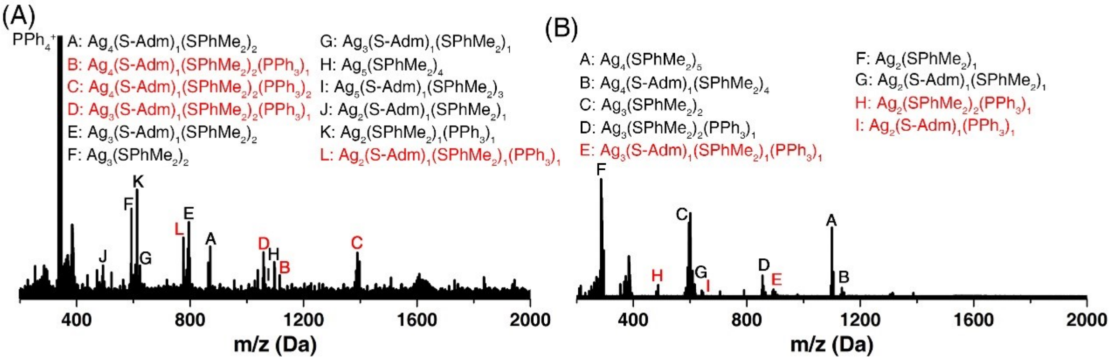

Fig. S7 ESI-MS of the raw solution of $Pt_1Ag_{24}(S\text{-}PhMe_2)_{18}$ that corresponding to the dark blue line in Fig. 2A, the sample of "30 min + PPh_4Br"): (A) detected in the positive mode; (B) detected in the negative mode. No other mass signal corresponding to nanocluster intermediates except for Pt_1Ag_{24} or Pt_1Ag_{28} signals (see Fig. 2) was observed over 2000 Da, probably because of the rapid transformation that the intermediates were hard to detect, or the instability of possible intermediates that would spontaneously transform into Pt_1Ag_{28} or Pt_1Ag_{24} nanoclusters. Besides, several PPh_3-containing small-sized Ag complexes have been detected, which originated from the transformation from $Pt_1Ag_{28}(S\text{-}PhMe_2)_x(S\text{-}Adm)_{18\text{-}x}(PPh_3)_4$ to $Pt_1Ag_{24}(S\text{-}PhMe_2)_{18}$.

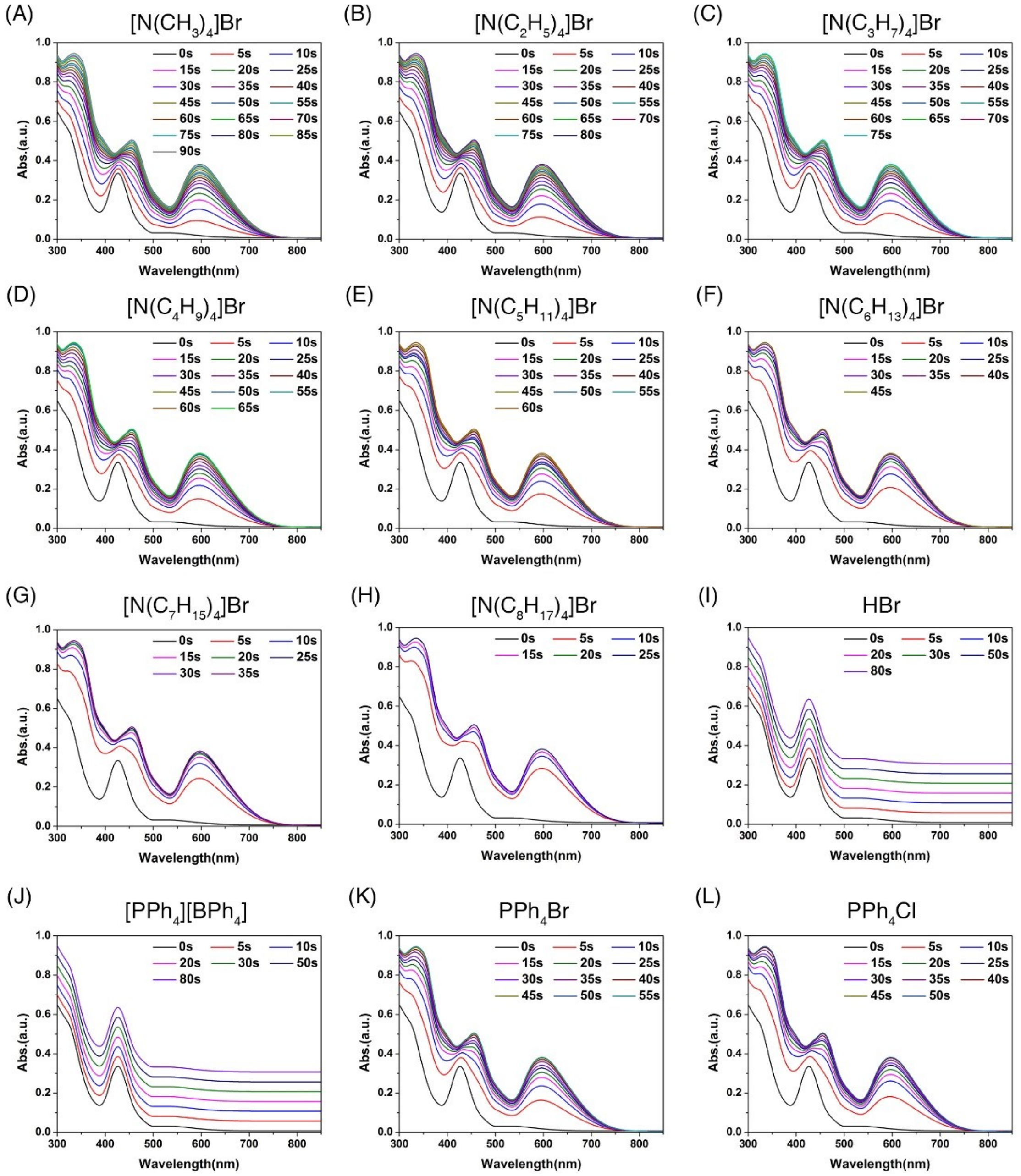

Fig. S8 Time-dependent UV-vis spectral of the conversion from $[Pt_1Ag_{28}(S\text{-}PhMe_2)_x(S\text{-}Adm)_{18\text{-}x}(PPh_3)_4]^{2+}$ to $[Pt_1Ag_{24}(S\text{-}PhMe_2)_{18}]^{2-}$ induced by the addition of $[N(C_mH_{2m+1})_4]^+Br^-$ (m = 1-8), HBr, $[PPh_4][BPh_4]$, PPh_4Br, or PPh_4Cl.

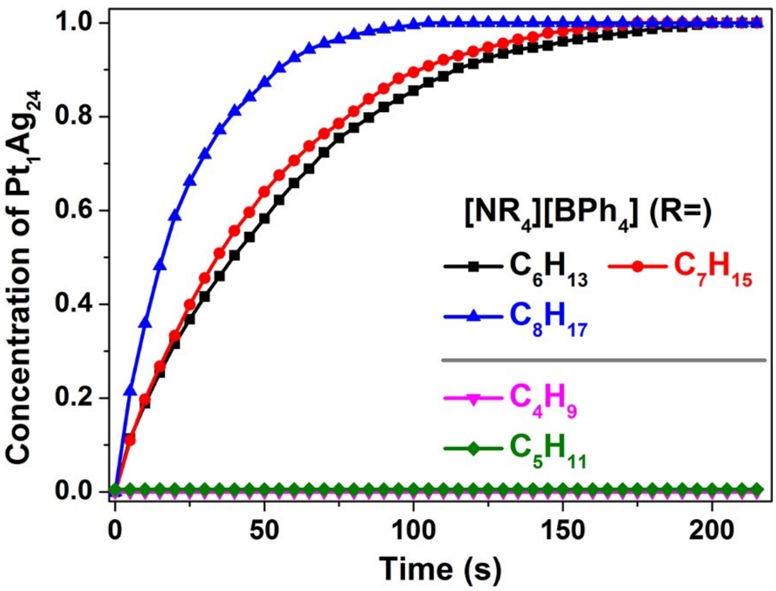

Fig. S9 Time-dependent concentration of the prepared $[Pt_1Ag_{24}(S\text{-}PhMe_2)_{18}]^{2-}$ induced by the addition of $[N(C_4H_9)_4]^+[BPh_4]^-$, $[N(C_5H_{11})_4]^+[BPh_4]^-$, $[N(C_6H_{13})_4]^+[BPh_4]^-$, $[N(C_7H_{15})_4]^+[BPh_4]^-$, and $[N(C_8H_{17})_4]^+[BPh_4]^-$ salts. Of note, the introduction of $[N(C_4H_9)_4]^+[BPh_4]^-$ or $[N(C_5H_{11})_4]^+[BPh_4]^-$ may transform the $[Pt_1Ag_{28}(S\text{-}PhMe_2)_x(S\text{-}Adm)_{18\text{-}x}(PPh_3)_4]^{2+}$ into $[Pt_1Ag_{28}(S\text{-}Adm)_{18}(PPh_3)_4]^{2+}$; however, $[Pt_1Ag_{28}(S\text{-}PhMe_2)_x(S\text{-}Adm)_{18\text{-}x}(PPh_3)_4]^{2+}$ and $[Pt_1Ag_{28}(S\text{-}Adm)_{18}(PPh_3)_4]^{2+}$ nanoclusters display a similar UV-vis characteristic, both with almost no absorption at 600 nm.
